# Contribution of Enzyme Catalysis to the Achievement of the United Nations’ Sustainable Development Goals

**DOI:** 10.3390/molecules28104125

**Published:** 2023-05-16

**Authors:** Dirk Holtmann, Frank Hollmann, Britte Bouchaut

**Affiliations:** 1 Institute of Process Engineering in Life Sciences, Karlsruhe Institute of Technology, Fritz-Haber-Weg 4, 76131 Karlsruhe, Germany; 2 Biocatalysis Group, Department of Biotechnology, Delft University of Technology, Van der Maasweg 9, 2629HZ Delft, The Netherlands; f.hollmann@tudelft.nl; 3 Safety and Security Science, Faculty of Technology, Policy and Management, Delft University of Technology, Jaffalaan 5, 2628BX Delft, The Netherlands; b.f.h.j.bouchaut@tudelft.nl

## 1. Introduction—The Sustainable Development Goals

In September 2015, the United Nations General Assembly established the 2030 Agenda for Sustainable Development, which includes 17 Sustainable Development Goals (SDGs). The interlinked SDGs are intended to be a ‘shared blueprint for peace and prosperity for people and the planet, now and in the future’ (https://sdgs.un.org/SDGs, accessed on 28 April 2023). The agenda emphasizes a holistic approach to achieving sustainable development for all, balancing the economic, social, and environmental dimensions of sustainable development. The agenda recognizes that ending poverty and other forms of deprivation must align with strategies that improve health and education, reduce inequality, and promote economic growth—all while tackling climate change and working to preserve our oceans and forests. Implementing the SDGs will require collaboration between different actors in government, industry, and civil society, as well as scientists from different disciplines. In the scientific community, the SDGs should provide a framework and serve as the guiding principles for research activity. Enzyme catalysis, among many other disciplines, can could represent a valuable contribution to the SDGs. The aim of this editorial chapter is to highlight the potential of enzyme catalysis in achieving the SDGs and to contribute to the realization of a ‘better world’, while reflecting on the deployment of these technologies to achieve these goals.

## 2. Relevance of Enzyme Technology to SDGs

For some of the SDGs, the relevance of enzyme technology is quite obvious, especially regarding the more technical goals. As enzyme catalysis is an integral part of the bioeconomy, its economic impact is also quite obvious. Enzyme catalysis is more indirectly related to social goals, such as SDG 1 (no poverty), SDG 4 (quality education), and SDG 5 (gender equality). In other words, every person, every scientist, and every research project should focus on achieving these goals.

Table 1 shows typical examples of the contributions of enzyme activities to the SDGs. It should be noted that our considerations relate to single enzymatic reactions or small reaction cascades. Biotechnological processes involving whole cells have broader implications for the SDGs. In addition, the information given in Table 1 is not exhaustive.

In addition, a number of optimizations can be applied to technically exploit these enzyme activities and improve processes in relation to the SDGs. These approaches include enzyme optimization to improve stability, to reduce the CO2 footprint of enzyme production and reactions, or to optimize reaction media, and thus, reduce the water requirements of enzyme reactions. The replacement of chemical reaction steps with enzyme catalysis represents a particularly important contribution of enzyme catalysis to the overall achievement of the SDGs.

## 3. Hurdles and Potentia l of Enzyme Catalysis in Achieving a ‘Better World’

Discussing the incentives and motives that influence the success rate and implementation possibilities of enzyme catalysis applications raises important questions about the role of technology in achieving the SDGs. While there is no doubt that the development of safer and more sustainable technologies is crucial to achieving these goals, researchers also risk placing too much faith in technology to solve complex problems. As already mentioned, achieving the SDGs requires collaboration between different actors in government, industry, and civil society, as well as scientists from different disciplines. Therefore, in biotechnology, approaches such as Responsible Research and Innovation (RRI, [36,37]), Value-Sensitive Design (VSD, [38,39]), and Safe (and sustainable)-by-Design (SbD, [40]) have been developed and implemented.

However, one of the challenges in developing sustainable technologies through the abovementioned approaches is defining what is ‘safe’ or ‘sustainable’. In the field of ‘green’ chemistry, emphasis is placed on designing products and processes that minimize or eliminate the use and generation of hazardous materials. However, it is important to consider the benchmark against which the ‘green’ alternative is compared. Developing ‘safer’ alternatives depends on these comparisons and the extent to which the associated emerging risks have been studied. For instance, short- and ultra-short PFAS (per- and polyfluoroalkyl substances) have been developed as a safer alternatives to long-chain PFAS as they undergo less bio-accumulation. However, due to their shorter C-chains, they are more mobile; thus, while they replace the type of hazard, they do not necessarily eliminate the risk [41]. Additionally, chemical compounds that are considered safe can turn out to have unexpected side-effects when released in nature. They can form other types of bonds and chemicals in reaction to compounds present in nature, e.g., by leaching into the environment [42]. For the further responsible development of enzyme catalysis, we must be aware of the occurrence of such instances in other domains and anticipate accordingly. Despite enzyme catalysis using ‘natural’ components, it can still give rise to emerging risks and uncertainties, which we want to avoid.

To this end, ‘good‘ stakeholder engagement is crucial. Actors from governmental bodies, industry, and civil society, and scientists from different disciplines, must collaborate to achieve inclusive design and incorporate specific values such as sustainability, equity, equality, and safety. Therefore, it is important to define who must be included and on what level. Does this inclusion pertain to the entire value chain, and if not, where does it ‘stop‘ [40]? Ideally, this inclusion should go beyond the product/process stage; however, considering a wide range of scenarios in which a broad scope of possible risks is evaluated is challenging, and would call for a different system set-up. Regarding the latter, economic motives and the substantial investments required for innovation often hinder the adoption of safer and more sustainable alternatives by conventional industry [43]. In other words, the current system places more weight on economic motives than on other important values such as sustainability and circularity, both of which are crucial to achieving the SDGs. If we want to achieve these and other climate-action goals, we cannot let economic motives or revenue continue to outweigh available alternatives that are safer, more sustainable, and/or circular. Regulatory bodies must promptly create incentives for industry to adopt and implement RRI, VSD, or SbD in their company regulations. Financial rewards can also stimulate industries to research and develop new, more sustainable alternatives. However, particularly if these rewards are derived from public money, we must be wary of whether the developed alternative is indeed safer/more sustainable/circular, and compared to what? If industries continue to fail to quickly adopt new, safer, more sustainable alternatives, enforcement may be necessary.

In order to avoid polarization in technological development and in stimulating and rewarding research and development, it would be beneficial to consider a higher-level abstract solution, whether it is a first- or second-order problem. We should ask: Is this really the solution or will it just provide a solution to part of the larger problem? We should be critical and not put too much faith in technology to solve all of our problems. Perhaps we should radically rethink our current ways of achieving our goals. Again, we should ask: Who is responsible for this, at what level, and who should be involved? These are complex questions, but discussing them will help develop insights into relevant values, arguments, and stakeholder involvement. Additionally, such insights will, in turn, contribute to aligning technological development with the SDGs and other climate goals.

In conclusion, discussing the incentives and motives that influence the success rate and implementation possibilities of enzyme catalysis applications highlights the need for critical reflection on technology and its role in contributing to the SDGs. We must not solely rely on technology to solve complex problems. However, if technological development is considered a necessity, and safer, more sustainable alternatives are available, we must ensure that these are adopted and are not hindered by economic motives. The urgency of ‘our’ global problems should take higher priority than a company’s revenue. This calls for a different system set-up; a transfer from a cost economy to a value economy in which conventional industry does not assign weight to values anymore, but higher-level discourse determines the directions taken.

## 4. Summary

In summary, the SDGs cannot be achieved without the contribution of enzyme catalysis. However, one must remain critical about what is to be gained and the benchmark with which the comparison is made. Is it really ‘greener”, more sustainable, and safer? However, in this field, it is also of utmost importance that the industry is ready to implement such technology, even if they require substantial financial investments. If not, it is important that government institutions either stimulate or enforce the transition.

## Figures and Tables

**Table 1:** Examples of enzyme activities and their relation to the SDGs (most of the enzymatic applications have an impact on different SDGs; here, the effects are shown exemplarily).

SDG 2: zero hunger	Enzymatic transformation of non-food biomass to starch so that there is no competition on farmland between food production and the production of chemicals and materials for other uses [1] Enzymatic production of fertilizer secures nutrition for the world’s growing population [2,3]
SDG 3: good health and well-being	Enzymatic synthesis of APIs enables shorter synthesis and products of higher quality compared to chemical processes [4,5] Tailored food, such as polyunsaturated fatty acids (PUFA), and enriched fats and oils [6] to improve food composition
SDG 6: clean water and sanitation	Enzymatic waste water treatment [7,8] to remove toxic substances Enzyme-based biosensors to detect pesticides and heavy metal toxicants in water [9,10,11]
SDG 7: affordable and clean energy	Use of enzymatic fuel cells to generate electrical energy [12,13] Improved production of biofuels through saccharification of non-food biomass [14,15]
SDG 8: decent work and economic growth	Opportunities for new businesses based on innovative biotechnological processes [16]
SDG 9: industry, innovation, and infrastructure	Production of high-value chemicals from non-food biomass [17,18] or conversion of alternative raw materials to intermediates for the chemical industry [19] Enzymatic catalysis as an alternative (more efficient) production route to chemical catalysis [20] and as an essential tool for cost-effective and sustainable pharmaceutical manufacturing [21]
SDG 11: sustainable cities and communities	Valorization of urban waste streams into value-added products [18,22]
SDG 12: responsible consumption and production	Enzyme catalysis involves greener synthesis, consuming fewer resources and generating less waste [23,24,25] The advantages of enzymes can be transferred to technical applications by means of process intensification, such as the integration of processes, optimized solvents, and alternative methods of energy transfer [26]
SDG 13: climate action	Enzymatic and energy-efficient CO_2_ conversion to reduce greenhouse gases [27,28]
SDG 14: life below water	Synthesis of biodegradable polymers [29] and enzymatic degradation of polyethylene terephthalate (PET) degradation [30,31] to prevent marine pollution.
SDG 15: life on land	Protecting crops with enzymatic pest control can help preserve biodiversity [32] Enzyme activity can be used to monitor soil degradation and, more importantly, to help restore soil [33,34,35]
